# MEFA (multiepitope fusion antigen)-Novel Technology for Structural Vaccinology, Proof from Computational and Empirical Immunogenicity Characterization of an Enterotoxigenic *Escherichia coli* (ETEC) Adhesin MEFA

**DOI:** 10.4172/2157-7560.1000367

**Published:** 2017-08-24

**Authors:** Qiangde Duan, Kuo Hao Lee, Rahul M Nandre, Carolina Garcia, Jianhan Chen, Weiping Zhang

**Affiliations:** 1 Department of Diagnostic Medicine/Pathobiology, Kansas State University College of Veterinary Medicine, Manhattan, KS 66506, USA; 2 Department of Biochemistry and Molecular Biophysics, Kansas State University, Manhattan, KS 66506, USA

**Keywords:** MEFA (multiepitope fusion antigen), Structural vaccinology, ETEC (enterotoxigenic *Escherichia coli*), Multivalent vaccine, Diarrhea

## Abstract

Vaccine development often encounters the challenge of virulence heterogeneity. Enterotoxigenic *Escherichia coli* (ETEC) bacteria producing immunologically heterogeneous virulence factors are a leading cause of children’s diarrhea and travelers’ diarrhea. Currently, we do not have licensed vaccines against ETEC bacteria. While conventional methods continue to make progress but encounter challenge, new computational and structure-based approaches are explored to accelerate ETEC vaccine development. In this study, we applied a structural vaccinology concept to construct a structure-based multiepitope fusion antigen (MEFA) to carry representing epitopes of the seven most important ETEC adhesins [CFA/I, CFA/II (CS1–CS3), CFA/IV (CS4–CS6)], simulated antigenic structure of the CFA/I/II/IV MEFA with computational atomistic modeling and simulation, characterized immunogenicity in mouse immunization, and examined the potential of structure-informed vaccine design for ETEC vaccine development. A tag-less recombinant MEFA protein (CFA/I/II/IV MEFA) was effectively expressed and extracted. Molecular dynamics simulations indicated that this MEFA immunogen maintained a stable secondary structure and presented epitopes on the protein surface. Empirical data showed that mice immunized with the tagless CFA/I/II/IV MEFA developed strong antigen-specific antibody responses, and mouse serum antibodies significantly inhibited *in vitro* adherence of bacteria expressing these seven adhesins. These results revealed congruence of antigen immunogenicity between computational simulation and empirical mouse immunization and indicated this tag-less CFA/I/II/IV MEFA potentially an antigen for a broadly protective ETEC vaccine, suggesting a potential application of MEFA-based structural vaccinology for vaccine design against ETEC and likely other pathogens.

## Introduction

Heterogeneity of enterotoxigenic *Escherichia coli* (ETEC) bacterial virulence factors is a major challenge for vaccine development. ETEC bacteria that produce adhesins to attach to different host receptors and enterotoxins to disrupt fluid homeostasis in small intestinal epithelial cells, are a leading cause of diarrhea in children under the age of 5 years in developing countries and in children and adults traveling from developed countries to ETEC endemic regions [1–3]. Currently, there is no licensed vaccine to protect against ETEC-caused children’s diarrhea or travelers’ diarrhea [4–7]. Because adhesin-mediated bacterial adherence to host cell receptors initiates ETEC infection, vaccines that induce antibodies preventing ETEC bacteria from adhering to host cells have been long regarded effective against ETEC infection.

Developing vaccines to prevent ETEC bacteria adherence and colonization, however, is hampered by the heterogeneity of ETEC bacterial adhesins. Different ETEC strains produce immunologically heterogeneous adhesins [8–10]. Antibodies derived from one type of adhesin may not block attachment of ETEC bacteria expressing different adhesins. The conventional approach by mixing together several live or killed strains that express a few different adhesins led to vaccine candidates that induce antibodies against homologous adhesins [11–14]. Recently, a novel strategy using reverse vaccinology and computer-aided structure-based multiepitope fusion antigen (MEFA) vaccine design has been explored to develop a safer and more effective ETEC vaccine.

The MEFA technology intends to design structure-defined and epitope-based immunogens to induce broadly protective antibodies against heterogeneous ETEC adhesins [15,16], facilitating the development of broad-spectrum ETEC vaccines. We recently constructed 6xHis-tagged adhesin MEFA CFA/I/II/IV, by integrating epitopes (*in silico* predicted) from the major subunits of the seven most important ETEC adhesins [CFA/II (CS1, CS2, CS3) and CFA/IV (CS4, CS5, CS6)] into a single MEFA protein [15]. Although that MEFA immunogen was shown to induce antibody responses to all seven ETEC adhesins, the 6xHis-tag (six histidines) carried by the recombinant MEFA immunogen may alter protein biochemistry properties [17]. Poly-histidine tag may also induce anti-histidine antibodies against histidine to cause potential adverse effects to human health, thus his-tagged antigens are considered less desirable for human vaccines. Additionally, antigenic structure of that 6xHis-tagged MEFA protein was not characterized.

Immunogen structure and antigenicity can be characterized empirically and also computationally. Recent advance in computational modeling and structural biology allows to assess structure properties of immunogens and to accelerate vaccine development [18,19]. In the current study, we cloned the CFA/I/II/IV MEFA gene without the 6xHis-tag, applied computational modelling to *in silico* characterize the antigenic structure of a tag-less CFA/I/II/IV MEFA, and examined immunogenicity of the new MEFA antigen in mouse immunization. In addition, we examined computational data and empirical data for immunogenicity congruence to assess potential application of computation simulation for structure-based ETEC vaccine development, likely proof of concept of MEFA application in structural vaccinology.

## Materials and Methods

### Construction of tag-less CFA/I/II/IV MEFA

The tag-less CFA/I/II/IV MEFA chimeric gene was PCR amplified from 6xHis-tagged CFA/I/II/IV MEFA plasmid DNA [15] with primers CFANcoI-F (5′-catgccatggaaatggctagcgcagtagaggat-‘3; NcoI site underlined) and T7-R (5′-tgctagttattggtcaggggt-‘3). PCR products were purified, digested with NcoI and EagI restriction enzymes (New England BioLabs, Ipswich, MA), and ligated into expression vector pET28α (Novagen, Madison, WI). The NcoI restriction site is located at the upstream of the 6xHis-tag region in vector pET28α, thus the new CFA/I/II/IV MEFA chimeric gene should not carry his-tag nucleotides. The cloned tag-less MEFA gene was DNA sequenced.

### Computational modeling of the tag-less CFA/I/II/IV MEFA protein

Program Rosetta [20–22] was used to generate an initial structure for the tag-less CFA/I/II/IV MEFA protein based on amino acid sequence, with the structure of CFA/I major subunit CfaB (PDB ID 3F85) [23] as the template. The fragment-based library was used to model segments of the tag-less MEFA that did not align with the template and to connect these segments to the aligned segments. A total of 50 comparative models were generated. The one with the top conformer score was selected as the final model, with each representing epitope specifically highlighted.

### Atomistic molecular dynamics simulation

Atomistic molecular dynamics (MD) simulations were performed in CHARMM [24], using CHARMM 36 force field [25] to further relax homology models and to investigate secondary structure and dynamics of the tag-less MEFA protein. Protein model was first solved in a cubic box of TIP3P water [26], and the total protein charge was neutralized by adding sodium ions. The final box size was about 69 Å. After energy minimization, 5.0 ns (nanosecond) simulation was used to equilibrate the structure by gradually reducing the harmonic positional restrain imposed on the protein backbone. The final production simulation course lasted 350 ns. Langevin dynamics [27,28] were performed at a constant temperature of 298 K and pressure of 1.0. SHAKE algorithm [29] was applied to maintain the length of all hydrogen-containing bonds and to allow of 2.0 fs (femto second) timestep. Particle mesh Ewald [30] was utilized for electrostatics with a real-space cutoff of 13 Å. Van der Waals interactions were gradually switched off between 12 Å and 13 Å.

### Tag-less CFA/I/II/IV MEFA protein expression and detection

*E. coli* strain transformed with pET28α plasmid carrying the tagless CFA/I/II/IV MEFA gene was cultured to express the tag-less MEFA protein. Recombinant protein was extracted and refolded as described previously [16].

Refolded tag-less CFA/I/II/IV MEFA protein was examined in 12% sodium dodecyl sulfate polyacrylamide gel electrophoresis (SDS-PAGE) and immune blot assays as previously described [15]. Protein purity and integrity were assessed in SDS-PAGE Coomassie blue staining and mass spectrophotometer under conditions of sinapinic acid (20 mg/ml) and a dilution of 50:50 with acetonitrile 0.1% trifluoroacetic acid (TFA).

### Mouse intraperitoneal (IP) immunization with tag-less CFA/I/II/IV MEFA protein

A group of 15 eight-week-old female BALB/c mice (Charles River Laboratories International, Inc., Wilmington, MA) was each intraperitoneally (IP) injected with 200 μg tag-less CFA/I/II/IV MEFA protein and 2 μg dmLT adjuvant (double mutant LT, LTR192G/L211A; provided by Walter Reed Army Institute of Research, Silver Spring, MD). IP route was used previously in mouse immunization with 6xHis-tagged CFA MEFA [15]. Each mouse received two booster injections with the same dose of the primary, at an interval of two weeks. A group of 15 mice without immunization were used as the control. Mice were sacrificed two weeks after the second booster. Mouse immunization study was approved by Kansas State University IACUC and supervised by a staff veterinarian.

To assess if removal of the 6xHis-tag affected immunogenicity of the MEFA protein, serum samples of the mice immunized with the newly constructed tag-less CFA/I/II/IV MEFA and of those previously immunized with the 6x His-tagged CFA/I/II/IV MEFA were comparatively examined.

### Mouse serum anti-adhesin IgG antibody titration

Serum samples from each immunized mouse and each control mouse were titrated for IgG antibodies specific to CFA/I, CS1, CS2, CS3, CS4 and CS5 in ELISAs as we previously described [15,31]. Antibodies specific to CS6 were not examined due to a lack of CS6 coating antigens. Mouse serum samples were two-fold diluted and examined in triplicate. Antibody titers were calculated from the highest serum dilution that produced OD readings of >0.3 above the background (highest dilution multiplies by adjusted OD) and presented in log_10_ [15,31].

### Mouse serum antibody adherence inhibition against adhesins CFA/I, CS1, CS2, CS3, CS4/CS6, CS5/CS6, and CS6

Serum samples from the immunized mice or the control mice were examined for *in vitro* antibody activities against bacterial adherence as previously described [15,31]. Briefly, ETEC bacteria expressing each CFA adhesin (3.5 × 10^6^ CFUs; MOI of five bacteria per cell) pre-treated with 10% mannose were mixed with 20 μl serum from the immunized or the control mice and incubated on a shaker (50 rpm) for 1 h at room temperature. The bacteria/serum mixture (brought to 300 μl with PBS) was added to each well of a 24-well tissue culture plate which contains Caco-2 cells (ATCC, #HTB-37TM, 7 × 10^5^ in confluent monolayer; in 700 μl cell culture medium) and incubated in a CO_2_ incubator (5% CO_2_) for 1 h at 37°C. After washes with PBS to remove non-adherent ETEC or *E. coli* bacteria, Caco-2 cells were dislodged with 0.5% triton X-100 (300 μl per well). Adherent ETEC or *E. coli* bacteria were collected by centrifugation (15,000 g for 10 min), suspended in 1 ml PBS, serially diluted, and plated on LB plates. Bacteria (CFUs) were counted after overnight growth at 37°C.

### Data Analysis

Protein dynamics simulation data analyses and structural visualization were performed using CHARMM [24], VMD [32] and R (http://www.R-project.org) programs. Protein secondary structure was calculated with STRIDE [33]. The solvent accessible surface area (ASA) was calculated by CHARMM with a water probe size of 1.4 Å. Relative ASA for each epitope was calculated using ASA of individual epitope normalized by the total MEFA protein ASA.

Mouse serum antibody titers expressed in log_10_ were analyzed using SAS for Windows, version 8 (SAS Institute, Cary, NC), with Student’s *t*-test for the significance of differences. Mouse serum antibody adherence inhibition activities were examined with non-parametric Mood’s Median Test at 95% confidence. Numeric results were presented as means and standard deviations. Calculated p values of less than 0.05 were considered significant when treatments were compared using two-tailed distribution and two-sample unequal variance.

## Results

### Tag-less CFA/I/II/IV MEFA protein was effectively expressed and extracted

**Figure 1:**
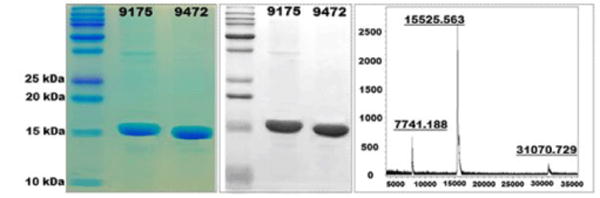
Tag-less CFA/I/II/IV MEFA protein expression and characterization. Left: SDS-PAGE Coomassie blue staining to show purity of refolded CFA/I/II/IV MEFA proteins. 9175, 6xHis-tagged CFA/I/II/IV MEFA (~17 kDa); 9472, tag-less CFA/I/II/IV MEFA (~15 kDa). Middle: Western blot with mouse anti-CFA/I antiserum to detect the refolded 6xHis-tagged or the tag-less CFA/I/II/IV MEFA protein. Right: Mass spectrophotometer to show the mass of the refolded tag-less CFA/I/II/IV MEFA protein.

Transformation of *E. coli* BL21 with plasmids carrying the tag-less CFA/I/II/IV MEFA gene yielded recombinant strain 9472. Strain 9472 expressed tag-less CFA/I/II/IV MEFA protein as effectively as strain 9175 expressed 6xHis-tagged MEFA. Tag-less CFA/I/II/IV MEFA protein was extracted at an average yield of 150 mg per liter culture (an average of five purifications) after refolding. Coomassie blue staining showed the tag-less MEFA protein was extracted at an estimated purity of over 95% (Figure 1).

Extracted protein was recognized by anti-CFA/I mouse antiserum (Figure 1). Matrix assisted laser desorption ionization-time of flight (MALDI-TOF) showed a predominant peak at a mass of 15,525 daltons for the tag-less CFA/I/II/IV protein (Figure 1), and 17,307 daltons for the 6xHis-tagged MEFA protein, the expected molecular mass for both proteins.

### Computational modeling showed that all seven representing epitopes were exposed on the MEFA protein surface

**Figure 2:**
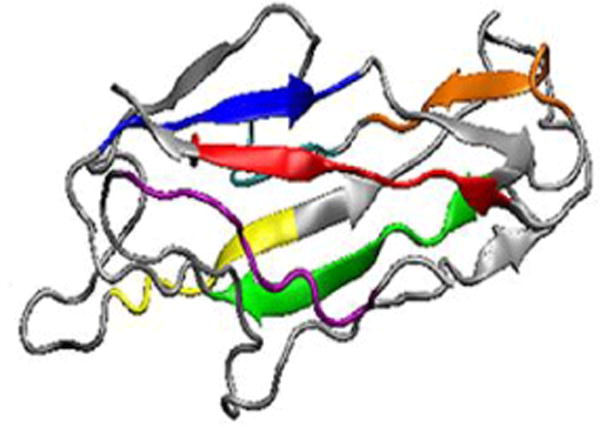
A computational model of the tag-less CFA/I/II/IV MEFA protein. Program Rosetta was used to construct protein model, with CFA/I major subunit CfaB model (PDB ID 3F85) as the template. Epitopes of the structural subunits of the seven adhesins were highlighted with colors: CFA/I (red), CS1 (orange), CS2 (yellow), CS3 (green), CS4 (blue) CS5 (purple), and CS6 (cyan; behind the red colored CFA/I epitope).

A total of 50 models were generated for the tag-less CFA/I/II/IV MEFA proteins. The one with the top conformer score showed a structure similar to backbone CFA/I CfaB subunit (Figure 2). Epitopes of the CFA/I, CS1, CS2, CS3, CS4, CS5 and CS6 in the tag-less MEFA protein were surface-exposed (Figure 2).

**Figure 3:**
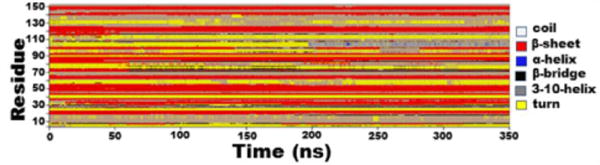
Atomistic molecular dynamics simulation to show the dynamic property of the tag-less CFA/I/II/IV MEFA peptide segments, measured with secondary structure evolution along time course. Program CHARMM with CHARMM 36 force field was used for molecular dynamics simulations. Residue on the y-axel indicated peptide segments of the tag-less CFA/I/II/IV MEFA. Time (ns; nanosecond) on the x-axel referred time of evolution from computational simulation. Colors represented different secondary structure: white, *E. coil*; red, β-sheet; blue, α-helix; black, β-bridge; gray, 3–10-helix; yellow, turn. Peptide segments retaining the same color along evolving time indicated secondary structure unchanged.

**Figure 4:**
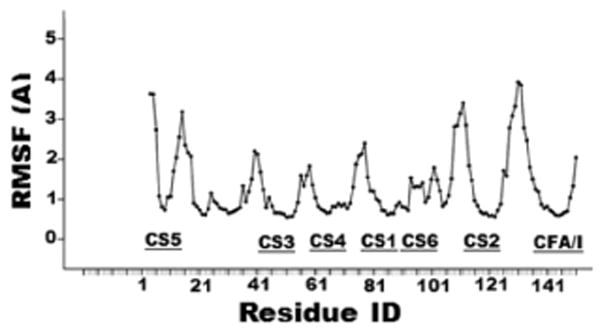
Molecular dynamic simulation to show the structure conformational flexibility of residues of the tag-less CFA/I/II/IV MEFA protein. Cα root-mean-square fluctuations (RMSF) on the y-axel indicated structure fluctuations or flexibility. Residue ID on the x-axel indicated amino acids of the CFA/I/II/IV MEFA, with representing epitopes marked.

Molecular dynamics simulation of protein secondary structural and dynamic properties showed the tag-less CFA/I/II/IVMEFA proteins maintained stable secondary structure during the entire simulation, indicated by peptide segments maintained same structure (the same color) as simulation time evolved (Figure 3). The root mean square deviation (RMSD) from the initial model gradually increased to 0.29 nm but became stabilized after 70 ns of simulations, indicating that the simulation reached the equilibrium. Variable root mean square fluctuation (RMSF) calculated to quantify conformational flexibility indicated that all seven epitope domains of the tag-less MEFA protein were stable during the simulation. Little dynamics was observed from the target epitope regions (Figure 4). This suggested that insertion of these epitopes did not appear to alter the stability of the overall structure of the backbone.

**Figure 5:**
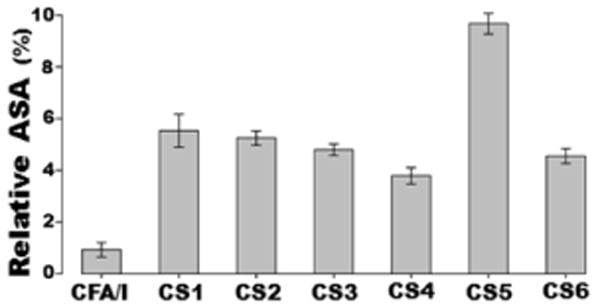
Averaged accessible surface areas (ASA) of representing epitopes of the tag-less CFA/I/II/IV MEFA, derived from atomistic molecular dynamics simulations. The error bars were standard deviations from different simulations.

Accessible surface area (ASA) analyses showed that all representing epitopes in the tag-less MEFA protein were surface exposed (Figure 5). ASA comparative studies indicated that the CS1 (5.5%), CS2 (5.3%), CS3 (4.9%), CS5 (9.7%) and CS6 (4.6%) epitopes were relatively more exposed.

**Figure 6:**
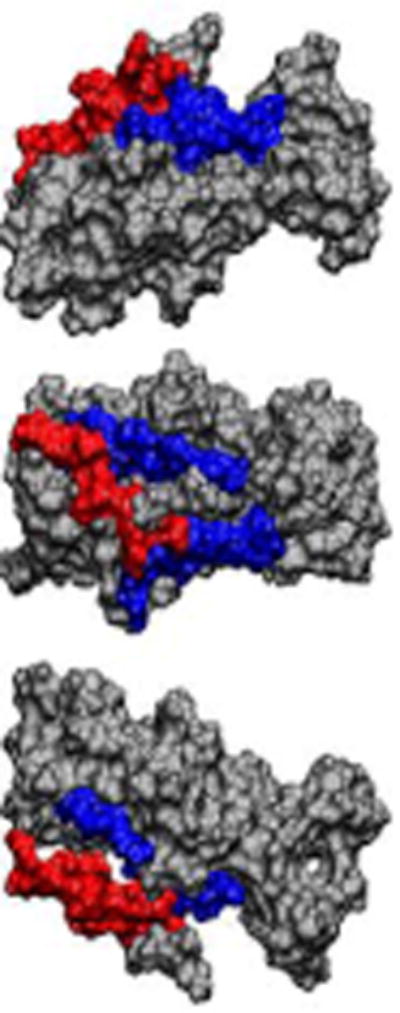
ASA to show CS5 epitope (in red) was flexible in the tagless CFA/I/II/IV MEFA. CS5 epitope (red colored) lay outside of two adjacent segments (blue colored). The adjacent segments are from residue number 27 to 35 (blue in the top row) and 141 to 149 (blue in the bottom row).

In contrast, the CAF/I epitope (0.93%) and CS4 epitope (3.8%) had a lower solvent accessibility (Figure 5). The CS5 epitope which located near the N-terminus showed more exposure as it lay on the outside of two adjacent peptide domains (Figure 6).

### Immunized mice developed antibody responses to target adhesins

**Figure 7:**
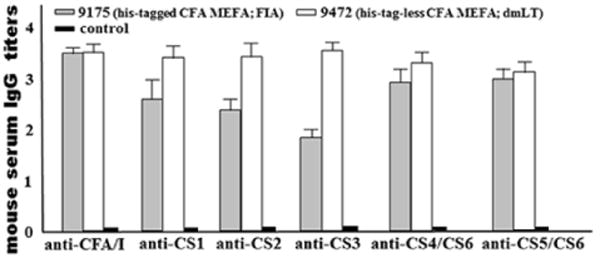
Mouse serum IgG antibody titers (log_10_) to CFA/I, CS1–CS6 adhesins induced from IP administered tag-less CFA/I/II/IV MEFA. Boxes in gray were IgG titers from mice IP immunized with the 6xHis-tagged CFA/I/II/IV MEFA (9175) and Freund’s incomplete adjuvant. Boxes in white were IgG titers from the mice IP immunized with the tag-less CFA/I/II/IV MEFA (9472) and dmLT adjuvant. Boxes in black were IgG titers from the control mice (no antibodies specific to these seven adhesins were detected). Bars indicated standard deviations of IgG titers from individual mice in teach immunized group or the control group.

Mice IP immunized with the tag-less CFA/I/II/IV MEFA protein developed antigen-specific antibodies (Figure 7). Anti-CFA/I, -CS1, -CS2, -CS3, -CS4/CS6 and anti-CS5/CS6 IgG antibody titers in serum samples of the immunized mice were 3.5 ± 0.15, 3.4 ± 0.25, 3.4 ± 0.27, 3.5 ± 0.20, 3.3 ± 0.23 and 3.1 ± 0.20 (log_10_). No antibodies were detected to these adhesins from the control mouse serum samples.

Compared to mice IP immunized with the 6xHis-tagged CFA/I/II/IV MEFA with Freund’s adjuvant [15], mice immunized with the tag-less CFA/I/II/IV MEFA and dmLT adjuvant developed significantly greater serum IgG responses to CS1, CS2, CS3 and CS4 (Figure 7).

### Serum samples from the immunized mice inhibited adherence of CFA/I, CS1, CS2, CS3, CS4/CS6, CS5/CS6, or CS6 adhesin

The serum samples pooled from the mice immunized with the tagless MEFA significantly inhibited adherence of ETEC or *E. coli* bacteria expressing CFA/I, CS1, CS2, CS3, CS4/CS6, CS5/CS6, or CS6 to Caco-2 cells, compared to the control mouse serum samples (Table 1).

## Discussion

Structure-based vaccine design or structural vaccinology aided by computational modeling and atomistic simulation provides a new tool to overcome antigen heterogeneity challenge in vaccine development [18,34–40]. For ETEC vaccine development, heterogeneity of ETEC bacterial virulence factors remains the key challenge. Different ETEC bacteria produce immunologically heterogeneous adhesins and enterotoxins. ETEC bacteria expressing any one or two types of these adhesins (over 23 ETEC adhesins have been identified) and either toxin (heat-labile toxin-LT or heat-stable toxin-STa) can cause diarrhea. Therefore, only vaccines inducing broad immunity against these adhesins and/or toxins are expected effective against ETEC [4,5]. Conventional vaccine candidates mixing together of a few live or killed strains induce immunity against homologous adhesins [11,13]. Excessive somatic antigens particularly harmful LPS carried by these cocktail products, however, could link to side effects, lower immune responses, and unsatisfied protection against ETEC diarrhea [5,7]. Instead of combining different bacteria strains, structure-based technology allows to include representative antigenic elements or epitopes from various ETEC virulence factors into a single MEFA immunogen for precision and broad immunogenicity. *In silico* structure data, however, are more of prediction at present; validation from empirical data is still considered essential for structural vaccinology [41,42]. The current study demonstrated that structural vaccinology helped to characterize structure and immunogenicity of the ETEC MEFA immunogen, and showed congruence between computational data and the empirical mouse immunization data at MEFA immunogenicity. That suggests the feasibility of applying structural vaccinology to assist ETEC vaccine development.

This tag-less CFA/I/II/IV MEFA was constructed by: 1) *in silico* predicting B-cell epitopes of the major structural subunits of the seven most important ETEC adhesins (CFA/I, CS1–CS6), 2) selecting one subunit (CFA/I subunit CfaB in this study) as the backbone at the criteria that this backbone subunit is very stable and relatively small-sized but carries multiple discontinuous and well-separated epitopes, 3) substituting the less antigenic epitopes of CfaB backbone with the most antigenic epitopes of the heterogeneous CS1–CS6 major structural subunits, and 4) computational modeling to optimize epitope substitution for a stable-structured MEFA. Computational modeling from the current study indicated all seven representing epitopes in the tag-less CFA/I/II/IV MEFA were surface exposed and presented at the β-sheet or the extension coil. Molecular dynamics simulation observed a low level of dynamics for these epitopes in the MEFA immunogen, suggesting these epitopes were stably presented by this MEFA protein. That correlated to the robust immune responses to each adhesin in the mice immunized with this CFA/I/II/IV MEFA.

Current data revealed removal of 6xHis-tag appeared not to affect the CFA/I/II/IV MEFA at protein expression, protein structure and stability, and immunogenicity. Data showed the tag-less and 6xHis-tagged CFA/I/II/IV MEFA proteins were expressed and extracted at the same yield (about 150 mg per liter culture medium) and purity (greater than 95%, based on PAGE Coomassie blue staining and mass spectrophotometer). Molecular dynamics simulation suggested a stable structure for the tag-less or the 6xHis-tagged MEFA (data not shown for the 6xHis-tagged MEFA). This tag-less CFA/I/II/IV MEFA showed equally or more immunogenic than the his-tagged CFA/I/II/IV MEFA (Figure 7), although the enhanced immunogenicity exhibited by the tag-less CFA/I/II/IV MEFA could be resulted from dmLT adjuvant, since dmLT was demonstrated to be equally or more effective (compared to Freund’s adjuvant) to immunoregulate parenterally immunized ETEC antigens [43,44]. The 6xHis-tag typically sticks out at protein surface (designed for nickel ion attachment during protein purification process) and may affect the exposure of the adjacent epitopes, through such negative effect could be limited presumably because the his-tag consists of only six histidines and eleven other residues of expression vector pET28α. Data from the current study showed the tag-less CFA/I/II/IV MEFA displayed immunogenicity to each representing epitope computationally and empirically.

Despite computational antigenicity prediction and empirical immunogenicity data from mouse immunization exhibited congruence, some variations were observed between *in silico* predicted accessible surface area (ASA) and *in vitro* antibody protection against bacterial adherence. CS5 epitope was calculated with a significant greater ASA (9.7%), but antibody protection against adherence of CS5/CS6 ETEC bacteria was the least efficient (41.6%), compared to antibody adherence inhibition against ETEC bacteria expressing the other six adhesins (an average of 52.8%). The CS5 epitope located close to the N-terminus likely allows it to be more surface accessible, and also more fluctuated as shown from dynamics simulation. In contrast, CS6 epitope showed a moderate ASA (4.6%); but antibody inhibition against adherence of CS6 ETEC bacteria was the most effective (75%). Similarly, the CFA/I epitope showed a significantly low ASA (0.93%), yet antibody protection against adherence of CFA/I ETEC H10407 (50.5%) was not noticeably lower than antibody inhibition against adherence of the other adhesins. The disagreement between a low CFA/I epitope ASA and a strong antibody protection against CFA/I bacteria adherence is explainable since other epitopes of the CfaB backbone also induce anti-CFA/I antibodies to additively protect against adherence of the CFA/I adhesin. The inconsistency of a high CS5 ASA and a below-an-average antibody protection against adherence of CS5 adhesin could suggest that the CS5 epitope is immunodominant but not necessarily strongly neutralizing; whereas differences between an average CS6 ASA and a greatest protection against adherence of CS6 ETEC bacteria may indicate that the CS6 epitope is strongly neutralizing. That also indicates future *in vivo* studies including colonization studies using a suitable animal model and even a controlled human challenge model, as well as immunization studies using other routes will be needed.

Congruence at overall immunogenicity of this tag-less CFA/I/II/IV MEFA between computational data and empirical mouse immunization data suggest the potential application of structural vaccinology in ETEC vaccine development. Whether antigen immunogenicity congruence also occurs from data in human immunization studies will be revealed in future human volunteer studies. It should be noted that some inconsistency between the ASA predicted from molecular dynamics simulation and the *in vitro* antibody protection among two individual epitopes warrants further improvement of structural vaccinology including the prediction of neutralizing epitopes instead of immunodominant epitopes; in return, it may also validate the need of empirical studies to confirm computational data.

## Figures and Tables

**Table 1:** Mouse serum antibody adherence inhibition assay results. Numbers of ETEC or *E. coli* bacteria that express CFA/I, CS1, CS2, CS3, CS4/CS6, CS5/CS6, or CS6 adhesin adhered to Caco-2 cells, after incubation with serum samples from the immunized mice or the control mice.

Mouse groups	Number of bacteria (×10^3^) adhered to Caco-2 cells						
	H10407 (CFA/I)	THK38/pEU405 (CS1)	DH5α/pEU588 (CS2)	E116 (CS3)	E106 (CS4/CS6)	UM75699 (CS5/CS6)	ETP98066 (CS6)
control	124 ± 7.9	64 ± 3.6	182 ± 6.8	347 ± 4.1	286 ± 4.4	436 ± 51.6	392 ± 8.7
immunized	77 ± 2.2 (p<0.01)	36 ± 3.3 (p<0.01)	85 ± 5.0 (p<0.01)	166 ± 5.0 (p<0.01)	162 ± 11.6 (p<0.01)	255 ± 10.7 (p<0.01)	96 ± 11.1 (p<0.01)
antibody adherence inhibition (%)	50.5	43.1	53	52.2	43.2	41.6	75
